# Differences in fractional amplitude of low-frequency fluctuations (fALFF) and cognitive function between untreated major depressive disorder and schizophrenia with depressive mood patients

**DOI:** 10.1186/s12888-024-05777-1

**Published:** 2024-04-24

**Authors:** Wensheng Chen, Jiaquan Liang, Xiangna Qiu, Yaqiao Sun, Yong Xie, Wenbo Shangguan, Chunguo Zhang, Weibin Wu

**Affiliations:** Department of Psychiatry, The Third People’s Hospital of Foshan, Foshan, 528000 Guangdong China

**Keywords:** Major depressive disorder, Schizophrenia with depressed mood, Fractional amplitude of low-frequency fluctuations, Gyrus rectus, Repeatable battery for the Assessment of Neuropsychological Status

## Abstract

**Background:**

Distinguishing untreated major depressive disorder without medication (MDD) from schizophrenia with depressed mood (SZDM) poses a clinical challenge. This study aims to investigate differences in fractional amplitude of low-frequency fluctuations (fALFF) and cognition in untreated MDD and SZDM patients.

**Methods:**

The study included 42 untreated MDD cases, 30 SZDM patients, and 46 healthy controls (HC). Cognitive assessment utilized the Repeatable Battery for the Assessment of Neuropsychological Status (RBANS). Resting-state functional magnetic resonance imaging (rs-fMRI) scans were conducted, and data were processed using fALFF in slow-4 and slow-5 bands.

**Results:**

Significant fALFF changes were observed in four brain regions across MDD, SZDM, and HC groups for both slow-4 and slow-5 fALFF. Compared to SZDM, the MDD group showed increased slow-5 fALFF in the right gyrus rectus (RGR). Relative to HC, SZDM exhibited decreased slow-5 fALFF in the left gyrus rectus (LGR) and increased slow-5 fALFF in the right putamen. Changes in slow-5 fALFF in both RGR and LGR were negatively correlated with RBANS scores. No significant correlations were found between remaining fALFF (slow-4 and slow-5 bands) and RBANS scores in MDD or SZDM groups.

**Conclusions:**

Alterations in slow-5 fALFF in RGR may serve as potential biomarkers for distinguishing MDD from SZDM, providing preliminary insights into the neural mechanisms of cognitive function in schizophrenia.

**Supplementary Information:**

The online version contains supplementary material available at 10.1186/s12888-024-05777-1.

## Background

Major depressive disorder (MDD) and schizophrenia with depressed mood (SZDM) are recognized as two distinct mental disorders. Nevertheless, they demonstrate a substantial overlap in clinical symptoms and features, especially during the early stages of the illnesses [1, 2]. Distinguishing between individuals with MDD and those with SZDM, considering behavioral manifestations, emotional processing, and cognitive functional impairment, poses a challenge [3].

In individuals with MDD, clinical symptoms extend beyond emotional disturbances, often manifesting as cognitive functional impairments [4]. Meta-analytic findings consistently indicate a moderate decline in cognitive functioning among individuals with MDD [5, 6]. Compared to healthy controls (HC), individuals with MDD exhibit reduced performance across various cognitive domains, including information processing speed, working memory, language acquisition, memory, visual spatial learning, and more [7–9]. In contrast, individuals with schizophrenia (SCZ) demonstrate a broader range of executive and cognitive deficits, encompassing cognitive flexibility, attention, processing speed, and response inhibition, among other aspects [10, 11]. There is a strong association between cognitive impairment and the prognosis and symptoms of patients with SCZ and MDD. Studies have shown that changes in neuropeptides [12] and alterations in negative/disorganized symptoms [13] may help differentiate the severity of cognitive impairment between SCZ and MDD patients. Furthermore, evaluating cognitive functional deficits in these patients can enhance our understanding of the pathophysiological mechanisms underlying these disorders.

Among patients with schizophrenia, the proportion of those experiencing depressive episodes is increasing, surpassing 65% [14]. Research indicates an increased risk of suicide among SZDM patients [15, 16]. Multiple meta-analyses indicate that administering antidepressant medication to patients with SZDM has clear therapeutic benefits [17–19]. However, studies have confirmed that only a minority of SZDM patients receive antidepressant medication treatment [20]. Patients with SZDM and MDD exhibit similar depressive symptoms, making it crucial to differentiate between these two groups for appropriate medication selection. However, there is currently a lack of objective and accurate methods for such differentiation.

Since its initial introduction by Biswal and colleagues, resting-state functional magnetic resonance imaging (rs-fMRI) has undergone rapid and substantial development over the past few decades, gaining widespread popularity [21]. Due to its inherent task-independent nature and user-friendly operation, rs-fMRI is well-suited for investigating psychiatric disorders [22]. In recent years, resting-state functional magnetic resonance imaging (rs-fMRI) has become one of the most widely used brain imaging techniques, extensively applied to explore the neurobiological mechanisms of various mental disorders, including SCZ, MDD, and bipolar disorder, among others [23–26]. Within this technique, the Amplitude of Low Frequency Fluctuation (ALFF) of the whole-brain rs-fMRI signal has been widely used due to its high test-retest reliability, rendering it a dependable algorithm. ALFF reflects the spontaneous fluctuations in blood oxygen level-dependent (BOLD) signals within the low-frequency range (0.01–0.08 Hz) during the brain’s resting state [27], closely correlating with brain neural activity and holding significant physiological significance. ALFF can also be subdivided based on frequency bands, primarily including slow-4 (0.027–0.073 Hz) and slow-5 (0.01–0.027 Hz) bands. The fractional amplitude of low-frequency fluctuations (fALFF) has advantages compared to other FC methods as it is relatively less susceptible to motion artifacts and exhibits lower sensitivity to physiological noise compared to the older ALFF method. Additionally, fALFF demonstrates stability during fMRI treatments, maintaining consistent reliability over time, making it a potential biomarker, particularly suitable for long-term observation and research [28]. For example, fALFF is used to analyze the association between abnormal amygdala signals and cognitive impairment in patients with SCZ [29]. This method has been widely employed in studies involving patients with SCZ and individuals with MDD, enhancing sensitivity and specificity in assessing intrinsic brain activity [30–33]. Furthermore, focusing on the slow-4 and slow-5 frequency bands of fALFF can more sensitively reveal the characteristics of intrinsic brain activity [34].

The above statements highlight considerable using ALFF and fALFF metrics to measure resting-state brain activity in both individuals with MDD and individuals with SCZ. However, there is limited research investigating the differences in brain fALFF and cognitive function in individuals with these disorders. Both individuals with MDD and individuals with SZDM are characterized by impairments in the cognitive domain, with altered fALFF values in several brain regions compared to HC.

In this study, we hypothesize the presence of differences in whole-brain fALFF (slow-4 and slow-5 frequency bands) among individuals with MDD and individuals with SZDM with depressive symptoms. Furthermore, we aim to explore the relationship between altered fALFF and cognitive impairments, seeking potential neuroimaging markers for cognitive dysfunction in individuals with individuals with MDD and individuals with SZDM with depressive symptoms.

## Methods

### Participants

The subjects of the study were inpatients from the Third People’s Hospital of Foshan City (Foshan Mental Health Center) from July 2020 to September 2022. The effect size was calculated using the G-Power software (Effect size f = 0.8, α = 0.05, Power = 0.95, G-power software 3.1.9.7; Franz Faul, University of Kiel, Kiel, Germany, https://www.psychologie.hhu.de/arbeitsgruppen/allgemeine-psychologie-und-arbeitspsychologie/gpower), and a total sample size greater than 40 met the statistical requirements. This study recruited a total of 118 participants (30 with SZDM, 42 with MDD, and 46 HC). The patient group comprised individuals met the diagnostic criteria for depression as outlined in the fourth edition of the Diagnostic and Statistical Manual of Mental Disorders (DSM-IV) by the American Psychiatric Association. Clinical interviews and diagnosis were conducted by two or more attending psychiatrists to confirm the diagnosis of either MDD or SZDM (with a need to restrict the Hamilton Rating Scale for Depression [HAMD] score to clarify depression). For patients with SZDM, the HRSD-24 assessment method was used, with a cutoff value set at ≥ 8 [35].

The age range for the study subjects was between 18 and 60 years, without restrictions on gender. Education level was required to be at least primary school and above, they had to be of Han ethnicity, and right-handed. Additionally, the study specifies that participants must be drug-naïve or have not used mood stabilizers, antidepressants, or typical antipsychotic medications in the past month. Simultaneously, participants must not have any other comorbidities.

As a control group comprised of 46 healthy individuals matched in terms of age and gender with the patient group were recruited. All control group members underwent screening by professionals, with clinical psychologists using the Structured Clinical Interview for DSM-IV-TR Axis I Disorders - Patient Edition to confirm the absence of mental disorders.

All participants were subject to exclusion criteria, including: current pregnancy or lactation, presence of other major medical or neurological disorders, alcohol or substance abuse, or other contraindications to MRI scanning. The study was conducted in accordance with the principles of the Helsinki Declaration. The research protocol was approved by the Ethics Committee of the Third People’s Hospital of Foshan City (Foshan Mental Health Center) (Ethics approval number: FSSY-LS202109), and all enrolled subjects provided informed consent before participating in the study.

### Measures

Basic demographic information (age, gender, and education level) and detailed information about the patients’ conditions were obtained through interviews, including age of onset and family history of patients, supplemented by caregivers. Handedness was measured using the Oldfield Handedness Questionnaire to ensure that all participants were right-handed.

We used the Hamilton Rating Scale for Depression-24 (HRSD-24) to assess the depressive symptoms of all participants. The Positive and Negative Syndrome Scale (PANSS) was used to measure the psychopathology of individuals with SZDM. The Repeatable Battery for the Assessment of Neuropsychological Status (RBANS) was used to evaluate the cognitive function of all enrolled participants. RBANS, a cognitive test tool developed by Randolph in 1998, has been widely used for cognitive assessments in conditions like individuals with MDD and individuals with SZDM. It comprises five cognitive domains (with ten subtests), including Immediate Memory (Story Memory and List Learning), Visuospatial/Constructional, Language, Attention, and Delayed Memory [36]. The RBANS test offers advantages such as simplicity of operation, high sensitivity, short testing time, and minimal learning effects. It is well-suited for repeated assessments due to its lack of significant practice effects [33].

### Imaging data acquisition

The MRI data were obtained from a GE Signa Pioneer 3.0T magnetic resonance scanner at the Third People’s Hospital of Foshan (Foshan Mental Health Center), Guangdong Province. A high-resolution T1-weighted structural image was obtained using a 3D-T1WI sequence, and resting-state data were collected using a GRE-SS-EPI sequence. The data underwent preprocessing using DPABI_V6.1, including removal of the first 10 time points, slice timing correction, realignment, normalization, smoothing, covariate regression, and band-pass temporal filtering (0.01–0.08 Hz). The fALFF analysis employed fast Fourier transform (FFT) to calculate power spectra and compute the square root of the power spectrum within the 0.01–0.08 Hz range to obtain fALFF values. The fALFF value represents the ratio of the power spectrum within the low-frequency range to that within the entire frequency range [30]. Only the rs-fMRI signals within the filtered slow-5 (0.01–0.027 Hz) and slow-4 (0.027–0.073 Hz) frequency bands were retained for subsequent analysis. Data quality control was performed using DPABI’s Quality Control program, discarding data with head motion exceeding 2.5 mm or rotation angles exceeding 2.5°.

### Statistical analysis

Data analysis was performed using SPSS 20.0, employing ANOVA and chi-square tests to compare demographic and clinical variables among different groups. Post hoc comparisons were adjusted using LSD or Tamhane correction based on homogeneity of variance, with *P* < 0.05 indicating statistical significance. Resting-state functional magnetic resonance imaging (rs-fMRI) data were subjected to One-way ANOVA and two-sample T-tests using DPABI, with Gaussian random field theory correction applied. Age, gender, and head motion parameters were included as covariates to explore whole-brain fALFF (slow-5, slow-4) differences among individuals with MDD, individuals with SZDM, and HC. DPABI tools identified specific anatomical positions, and results were presented accordingly. Using age, gender, education level, and depression symptoms as covariates, extract significant fALFF values from altered brain regions and perform partial correlation analysis with RBANS scores. The results underwent Bonferroni correction. Statistical analysis results can assist doctors in more accurately assessing the effectiveness of treatment methods and guiding clinical decisions, thereby improving patient treatment outcomes and quality of life.

To assess the potential of modified fALFF values in distinguishing individuals with SZDM, MDD, and HC, we conducted receiver-operating characteristic curve (ROC) analysis. The area under the curve (AUC) serves as a metric for overall diagnostic accuracy, calculated using SPSS 20.0 software (SPSS Inc., Chicago, Illinois, USA).

## Results

### Demographic and clinical data

**Fig. 1:**
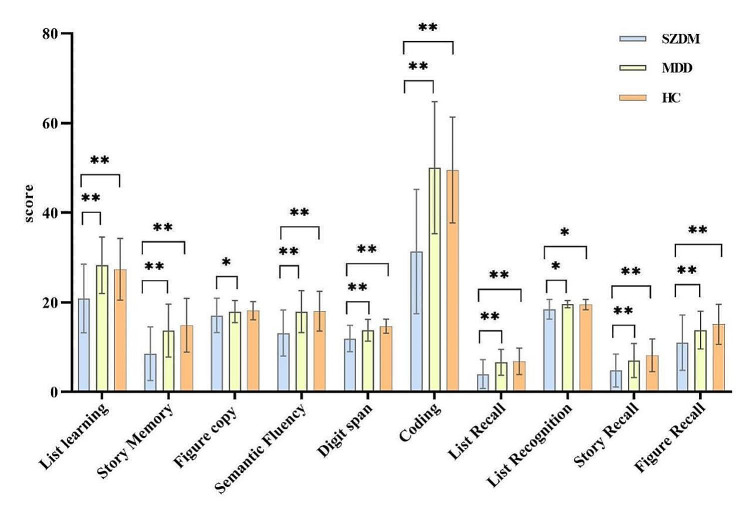
SZDM, MDD, and HC group RBANS scores, * indicating *p* < 0.05, ** indicating *P* < 0.001

This study included 118 participants and completed the collection of imaging data. Among them, there were 30 patients with SZDM, 42 patients with MDD, and 46 HC. The results indicate that there is no statistically significant gender difference among the three groups (*P* > 0.05). However, there are statistically significant differences in terms of family history of mental illness, age, education level, HDRS-24, and total PANSS score (*P* < 0.05). Detailed information is provided in Table 1. The correlation analysis of RBANS scores among the three groups is presented in Fig. 1.

**Table 1:** Demography and clinical characteristics

Variables	SZDM (30)	MDD (42)	HC (46)	F/χ^2^	*p*-value
Sex (female/male)	16/14	25/17	27/19	0.65	0.72
Age (years)	42.6 ± 8.6	26.71 ± 11.59	35.8 ± 12.8	17.65	< 0.001
Education (years)	9.83 ± 2.65	12.36 ± 2.31	12.1 ± 3.61	7.37	0.001
Family mental health history (None/Exist)	21/9	35/7	42/4	8.42	0.015
HDRS-24	11.87 ± 6.16	25.22 ± 5.27	2.50 ± 3.36	235.33	< 0.001
Total Score for PANSS	77.4 ± 16.60	30.64 ± 1.16	30.15 ± 0.56	353.94	< 0.001

MDD: Major Depressive Disorder; SZDM: Schizophrenia with Depressive Mood; HC: Healthy Control; HDRS-24: Hamilton Depression Rating Scale-24; PANSS: The Positive and Negative Syndrome Scale; Sex, Education, and Family mental health history are subjected to Chi-Square Test (χ2), while others are subjected to Analysis of Variance (F); *P*-value indicates the significance of the Analysis of Variance (F); Age, Family Mental Health History, and HDRS-24 are adjusted using Tamhane correction.

### Comparison of fALFF among MDD, SZDM, and HC

**Fig. 2:**
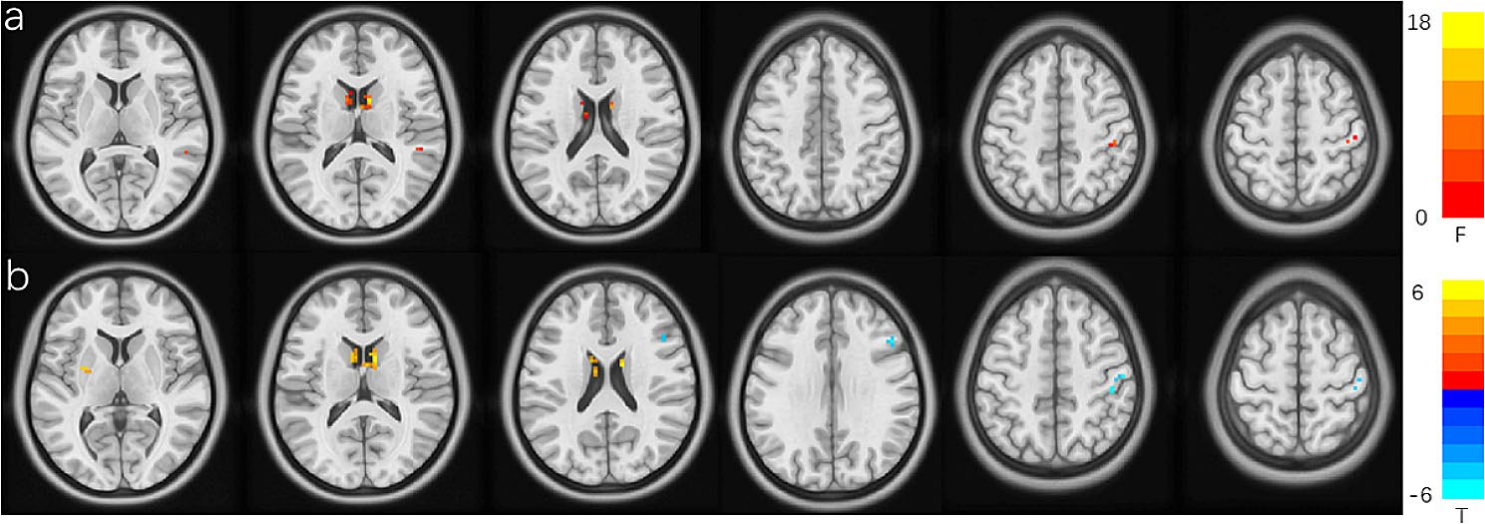
**(a)** Variance analysis of the standardized z-values of fractional amplitude of low-frequency fluctuations (zfALFF) in the slow-4 frequency band comparing MDD, SZDM, and HC. Highlighted colors indicate significantly different regions, corrected using GRF (Voxel *p* < 0.001, Cluster *P* < 0.05). **(b)** Comparison of slow-4 frequency band fALFF changes between the SZDM and HC groups, corrected using GRF (Voxel *p* < 0.001, Cluster *P* < 0.05). Yellow and blue represent regions where SZDM patients have higher and lower activity compared to HC, respectively

Through one-way ANOVA, significant differences were observed among the MDD, SZDM, and HC in multiple brain regions for slow-4 fALFF. Particularly, significant differences were observed in the Caudate_R, Caudate_L, Postcentral_R, and Temporal_Sup_R regions (Voxel *p* < 0.001, Cluster *P* < 0.05) following GRF correction. Subsequent post hoc analyses revealed that in the comparison between the SZDM and HC, the SZDM group demonstrated significantly increased fALFF in the right Putamen_R, Putamen_L, and Caudate_R regions, along with significant decreases in the Postcentral_R and Frontal_Inf_Tri_R regions. However, there were no significant differences were observed in the comparisons between the MDD group and the HC, as well as between the MDD group and the SZDM group. These findings remained non-significant after controlling for age, gender, and education level. Please refer to Table 2; Fig. 2 for detailed information.

**Fig. 3:**
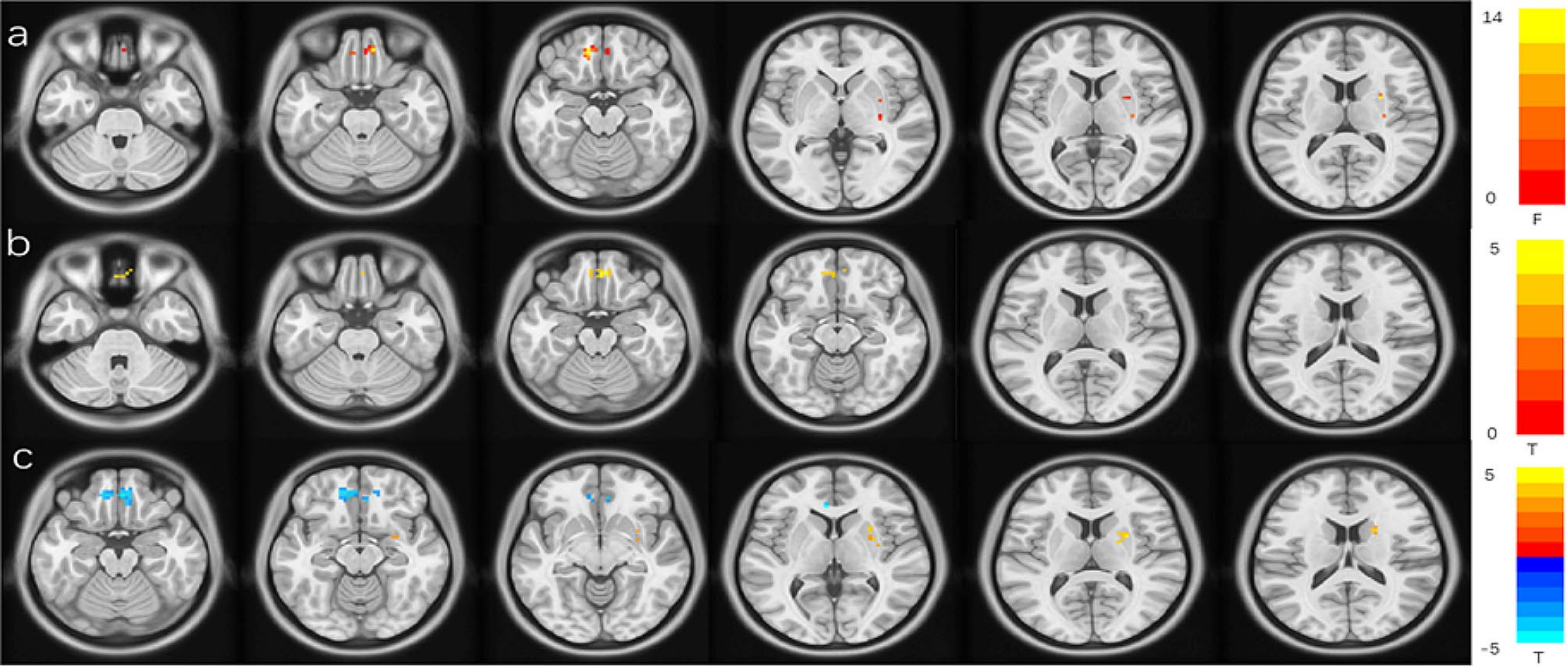
**(a)** Variance analysis of the standardized z-values of fractional amplitude of low-frequency fluctuations (zfALFF) in the slow-5 frequency band comparing MDD, SZDM, and HC. Highlighted colors indicate significantly different regions, corrected using GRF (Voxel *p* < 0.001, Cluster *P* < 0.05). **(b)** Regions of fALFF changes in the slow-5 frequency band comparing the MDD and SZDM groups, corrected using GRF (Voxel *p* < 0.001, Cluster *P* < 0.05). Yellow represents regions where MDD patients have higher activity compared to SZDM. **(c)** Regions of fALFF changes in the slow-5 frequency band comparing the SZDM and HC groups, corrected using GRF (Voxel *p* < 0.001, Cluster *P* < 0.05). Blue and yellow represent regions where SZDM patients have lower and higher activity compared to HC, respectively

Regarding slow-5 fALFF, ANOVA results indicated significant alterations in four brain regions, including Rectus_L, Frontal_Sup_Orb_L, Rectus_R, and Putamen_R (with GRF correction, Voxel *p* < 0.001, Cluster *P* < 0.05). In pairwise comparisons, no significant differences were observed in slow-5 fALFF between the MDD group and the HC. Comparison with the HC, the SZDM group showed significantly increased fALFF in the Putamen_R region and decreased fALFF in the Rectus_L, Rectus_R, and Frontal_Sup_Orb_L regions. In contrast, when comparing with the SZDM group, the MDD group exhibited significant increases in fALFF in the Rectus_L and Rectus_R regions. Similar to the previous analysis, adjustments for age, gender, and education level were performed. Refer to Table 3; Fig. 3 for detailed results.

**Table 2:** Differences in slow-4 between SZDM, MDD, and HC

Group comparison	Brain region	Cluster size	Peak (MNI)			T value
			X	Y	Z	
ANCOVA	Caudate_R	10	9	6	15	18.39
	Caudate_L	6	-9	6	15	12.19
	Postcentral_R	8	45	-30	54	15.02
	Temporal_Sup_R	5	54	-39	9	10.23
SZDMvs HC	Putamen_R	5	30	-21	-3	5.72
	Putamen_L	15	-27	-6	3	6.06
	Caudate_R	12	9	-3	18	5.85
	Postcentral_R	24	45	-27	51	-4.93

**Table 3:** Differences in slow-5 between SZDM, MDD, and HC

Group comparison	Brain region	Cluster size	Peak (MNI)			T value
			X	Y	Z	
ANCOVNA	Rectus_L	14	-12	45	-18	14.93
	Rectus_R	17	3	42	-21	14.85
	Putamen_R	7	27	3	12	14.11
MDD vs. SZDM	Rectus_R	15	3	42	-21	4.58
SZDM vs. HC	Rectus_L	33	-12	42	-18	-5.74
	Putamen_R	36	27	3	12	5.56

### The correlations between slow-4 fALFF and slow-5 fALFF values in abnormal brain regions and RBANS scores were examined

A correlation analysis was conducted between the slow-4 fALFF and slow-5 fALFF values in abnormal brain regions and RBANS scores (The correlation analysis between activated brain regions and cognitive scores can be found in Figure S1).

The results indicated a weak positive correlation between slow-4 fALFF values in the Caudate_R, Caudate_L, and Putamen_L brain regions and RBANS scores in language (semantic fluency), attention (symbol coding), figure recall, and story recall. However, these correlations did not achieve statistical significance after Bonferroni correction.

In the case of slow-5 fALFF, significant correlations were observed between fALFF values in the Rectus_R, Rectus_L and putamen_R brain regions and RBANS scores, and these correlations passed the Bonferroni correction. Refer to Figure S2 for specific results.

### ROC analysis in patients with SZDM, MDD and HC

**Fig. 4:**
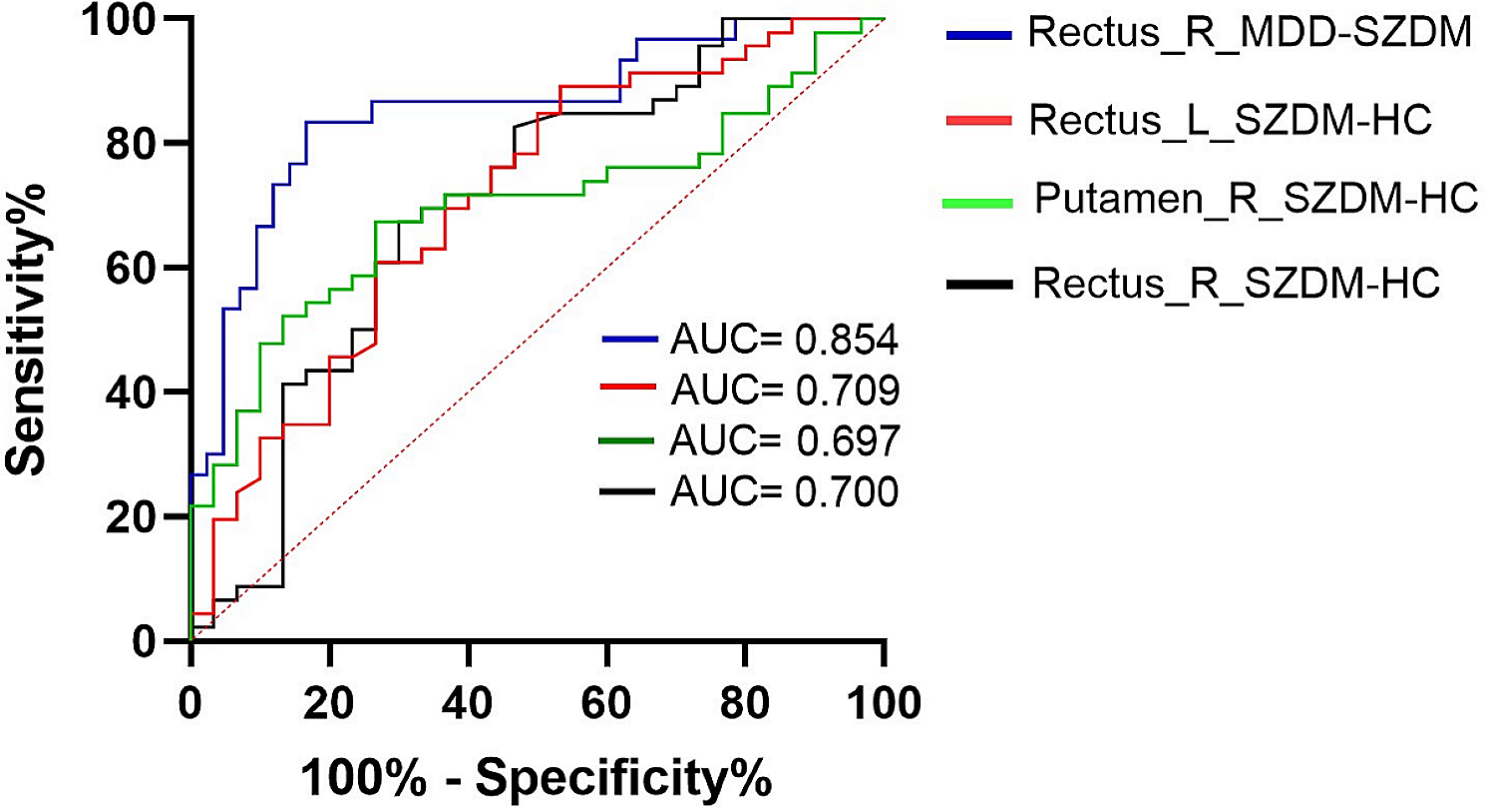
fALFF values across different brain regions and Receiver Operating Characteristic (ROC) curves for participants in different groups

When plotting the ROC curve, the false positive rate (1-specificity) is represented on the x-axis, and the true positive rate (sensitivity) on the y-axis, connecting all points on the graph (each corresponding to a cutoff level) to generate the ROC curve (see Fig. 2). The AUC values for Rectus_R MDD-SZDM, Rectus_L SZDM-HC, Putamen R SZDM-HC, and Rectus R SZDM-HC are 0.854, 0.709, 0.697, and 0.700, respectively (Fig. 4).

## Discussion

This study aimed to investigate fALFF alterations in the slow-5 and slow-4 frequency bands among individuals with MDD and SZDM. Significant differences in fALFF were observed in seven brain regions among individuals with MDD, SZDM, and HC. Individuals with SZDM and MDD patients exhibited cognitive impairments and fALFF differences when compared to HC. Notably, fALFF in Rectus brain regions exhibited a negative correlation with cognitive measures. These findings suggest that fALFF in the Rectus region, particularly in the slow-5 band, could be a marker for distinguishing cognitive differences between individuals with MDD and SZDM, enhancing our understanding of these disorders’ neural mechanisms and aiding clinical insights.

Previous research have reported changes in fALFF within regions such as the parahippocampal gyrus, temporal pole [37], hippocampus/parahippocampal gyrus [38], and temporal fusiform gyrus [39]. However, these anomalies were not observed in this study. These discrepancies could stem from cultural differences, population heterogeneity, medication status, and variations in statistical methods. Importantly, the majority of previous studies primarily employed the 0.01–0.08 Hz frequency range to depict brain activations. However, evidence suggests that spontaneous oscillatory features within the same neural network differ across frequency bands, and fALFF is more sensitive in detecting intergroup functional differences relative to ALFF [34]. Furthermore, research conducted by Huang et al. demonstrated that the investigation of abnormal low-frequency oscillations in individuals with SCZ is contingent on the frequency band [40]. Hence, in exploring intergroup differences between slow-5 fALFF and slow-4 fALFF bands, slow-5 fALFF might provide higher sensitivity and reliability.

In this study, we observed that in the context of slow-4 fALFF, patients with SZDM showed increased bilateral putamen, increased right caudate nucleus, and decreased right posterior cingulate cortex compared to the HC. Moreover, regarding slow-5 fALFF, individuals with MDD demonstrated increased right gyrus rectus (RGR), while patients with SZDM exhibited decreased left gyrus rectus (LGR) and increased right putamen compared to the HC. In line with previous research findings, the alterations in bilateral putamen, right caudate nucleus, and right posterior cingulate cortex are consistent with result from other studies [34, 41]. Nevertheless, the reduction in slow-4 fALFF values in the right frontal inferior triangular region observed in individuals with SCZ contradicts the findings of Wang et al. [42], possibly due to the coexistence of depressive symptoms in our study participants. The striatum, a vital component of the human brain responsible for motor control and reward processing, plays a pivota role in the study [34, 43]. As part of the striatum, the caudate nucleus is involved in various functions, including execution, motor functions, motivation, and emotions [41]. The gyrus rectus (GR), once considered a non-functional and primitive fold within the orbitofrontal cortex, has now gained recognition for its functional importance in recent studies. Research has even links central nervous system lesions in the GR to epilepsy seizures [44]. Located on the basal surface of the frontal lobe [45], the GR plays a critical role in memory, decision-making, and executive functions [39].

In our study, we found that, in the context of slow-5 fALFF, patients with SZDM exhibited decreased fALFF values in the rectus brain region compared to individuals with MDD and HC. This implies that individuals with SZDM exhibit poorer performance in domains related to memory, decision-making, and executive functions. Additional correlation analyses revealed negative associations between the rectus region and aspects of attention, delayed memory, language function, and immediate memory. This indirectly supports the idea that individuals with SZDM may display relatively inferior cognitive performance in these domains than individuals with MDD and HC. Researchers like Joo et al. have noted that surgical removal of the GR can temporarily impact memory recall and language function negatively [46]. Existing studies have indicated that asymmetry in the cortical thickness and surface area of GR could heightened challenges in social functioning, elevated risk of personality disorders, and diminished cognitive empathy abilities. Asymmetry in GR could potentially serve as a common neuro-correlational index linking neurotypical adult personality disorders, cognitive empathy, and social functioning. The asymmetry of the GR affects social functioning by influencing cognitive processes related to self and others’ emotions. Therefore, interventions that enhance leftward asymmetry might help improve social functioning in individuals with autism [47]. Furthermore, the gray matter volume of the RGR could potentially be a marker for distinguishing prodromal individuals with SCZ from HC [48].

In terms of cognitive function scores, both individuals with SZDM and those with MDD exhibited lower cognitive function compared to HC. More specifically, cognitive test scores of individuals with SZDM were significantly lower than those of individuals with MDD, which aligns with previous research and underscores the more severe cognitive impairments in individuals with SZDM. Individuals with MDD might affect reaction and decision times, as well as processing speed. Recent studies suggest that reaction times, decision times, and processing speed of individuals with MDD are slower than those of HC [49]. However, our study did not observe significant differences between individuals with MDD and HC in these aspects, potentially attributed to heterogeneity or variation in statistical methods. Concurrently, evidence suggests that individuals with SCZ have slower information processing speeds. Additionally, memory deficits could contribute to the decelerated information processing speed in these patients [50]. Some studies have reported a link between cognitive and functional impairments, as well as negative symptoms, in individuals with SCZ [51]. Numerous studies highlight the association between cognitive impairment and symptoms, as well as the prognosis of individuals with SCZ and MDD. However, cognitive impairment in individuals with SCZ is frequently considered irreversible due to its association with neural damage [52]. Hence, cognitive test analysis is of crucial importance in distinguishing between diagnoses of these two disorders.

In cognitive inhibition tests, patients with RGR removal performed poorly in the Stroop color-word test, while patients with GR removal showed inferior performance in the Trail Making B test compared to the HC. This finding suggests a distinct role of the medial orbitofrontal cortex in cognitive inhibition processes, with potential involvement in processes such as response inhibition and stimulus-based attention switching. Bilateral GR is considered a potential target for deep brain stimulation in individuals with treatment-resistant MDD [53]. Furthermore, studies have indicated the critical role of GR in effective communication [54]. Research by Kristine et al. identified GR as significant in inhibiting inappropriate behavior [55]. Georgiopoulos et al. reported an association between the GR and executive functions [56]. Prior literature has shown an association between Alzheimer’s disease and abnormal GR [57]. Research by Bahar Fuchs et al. uncovered notable differences in the GR region among patients with amnestic mild cognitive impairment compared to HC, supporting the hypothesis that abnormal GR might contribute to cognitive impairment to some extent [58]. Hence, researchers suggest that GR might be closely linked to cognitive and memory functions. In summary, both structurally and functionally, the GR is closely related to cognition, consistent with our study results. Based on these findings, the GR could serve as a potential marker to differentiate the cognitive impairment differences between individuals with MDD and those with SZDM. The association of the GR with cognitive impairment offers a fresh perspective for the clinical diagnosis and intervention of individuals with MDD and individuals with SCZ [59].

In general, in diagnostic tests, an AUC in the range of 0.5–0.7 indicates low diagnostic accuracy, an AUC in the range of 0.7–0.9 suggests moderate accuracy, and an AUC above 0.9 indicates high diagnostic accuracy [60]. The ROC results indicate that the increased fALFF values in RGR may serve as potential neuroimaging biomarkers for patients with SZDM and MDD.

Accurately distinguishing between SZDM and MDD patients will be crucial in guiding patient care and treatment choices. The elevation of fALFF values in RGR may serve as a potential neuroimaging biomarker for SZDM and MDD patients, representing a novel research direction.

This study also has several limitations. Firstly, the sample size is relatively small. As participants were recruited based on real-world scenarios, sample size calculation was not conducted at the outset of the study. Further multi-sample studies are needed in the future to validate the findings. Secondly, the cross-sectional nature of this study limits its ability to establish direct causal relationships between individual brain functional changes and cognitive impairments in MDD and SZDM patients. Post-treatment studies should be incorporated in future research to explore the effects of treatment on brain regions. Additionally, the similarity in symptoms between SZDM and MDD patients may confound the test results, necessitating larger samples to avoid this situation. In conclusion, larger longitudinal studies are necessary to reveal the potential brain mechanisms underlying the changes in brain function and cognitive impairments in individual MDD and SZDM patients.

## Conclusions

In summary, our study has pioneering significance as we used rs-fMRI to explore the differences in fALFF and cognition in untreated individuals with MDD and individuals with SZDM. Our findings suggest that the RGR could potentially serve as a cognitive marker for distinguishing between individuals with MDD and individuals with SZDM, providing preliminary evidence for understanding the neural mechanisms of cognitive function in SZDM.

### Electronic supplementary material

Below is the link to the electronic supplementary material.

Supplementary Material 1

## Data Availability

The datasets generated and/or analyzed during the current study are not publicly available due to privacy and ethical restrictions but are available from the corresponding author on reasonable request.
