# Supplementary material for: Differences in fractional amplitude of low-frequency fluctuations (fALFF) and cognitive function between untreated major depressive disorder and schizophrenia with depressive mood patients
Source: BMC Psychiatry. 2024 Apr 24;24:313. doi: 10.1186/s12888-024-05777-1 (PMC11044294; doi:10.1186/s12888-024-05777-1)
Supplement: Supplementary file 1 — Supplementary Material 1 [file 12888_2024_5777_MOESM1_ESM.docx]

**Differences in fractional amplitude of low-frequency fluctuations (fALFF) and cognitive function between untreated major depressive disorder and schizophrenia with depressive mood patients**

Wensheng Chen, Jiaquan Liang, Xiangna Qiu, Yaqiao Sun, Yong Xie, Wenbo Shangguan, Chunguo Zhang*, Weibin Wu *

Affiliation/address:

Department of Psychiatry, The Third People's Hospital of Foshan, Foshan, Guangdong 528000, China.

*Correspondence:

Chunguo Zhang

Department of Psychiatry, The Third People's Hospital of Foshan, Foshan, Guangdong 528000, China.

Email: cgzhang1994@163.com

Weibin Wu

Department of Psychiatry, The Third People's Hospital of Foshan, Foshan, Guangdong 528000, China.

Email: wuwb128@163.com


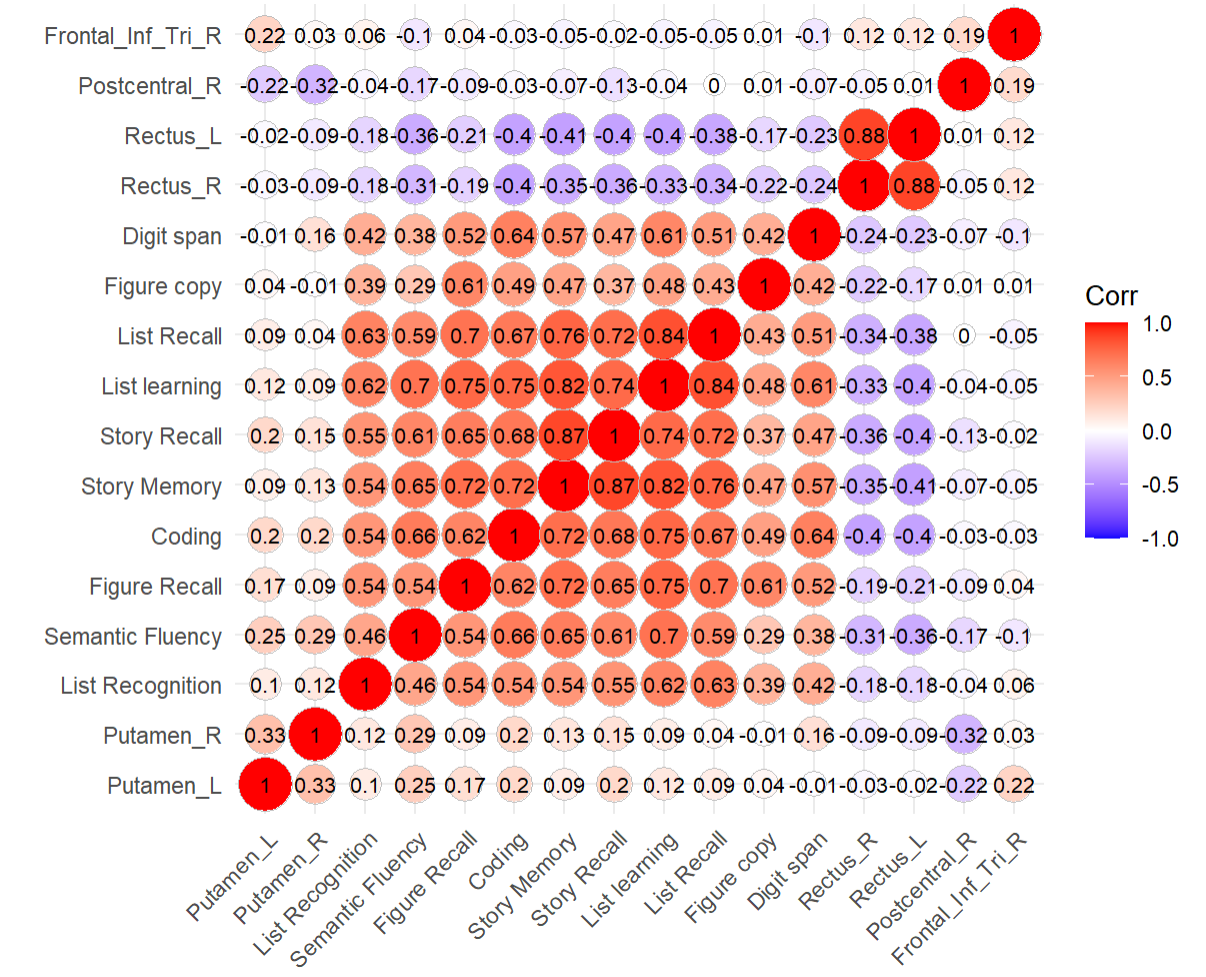


Figure S1: Heatmap of the correlation analysis between activated brain regions and cognitive scores.


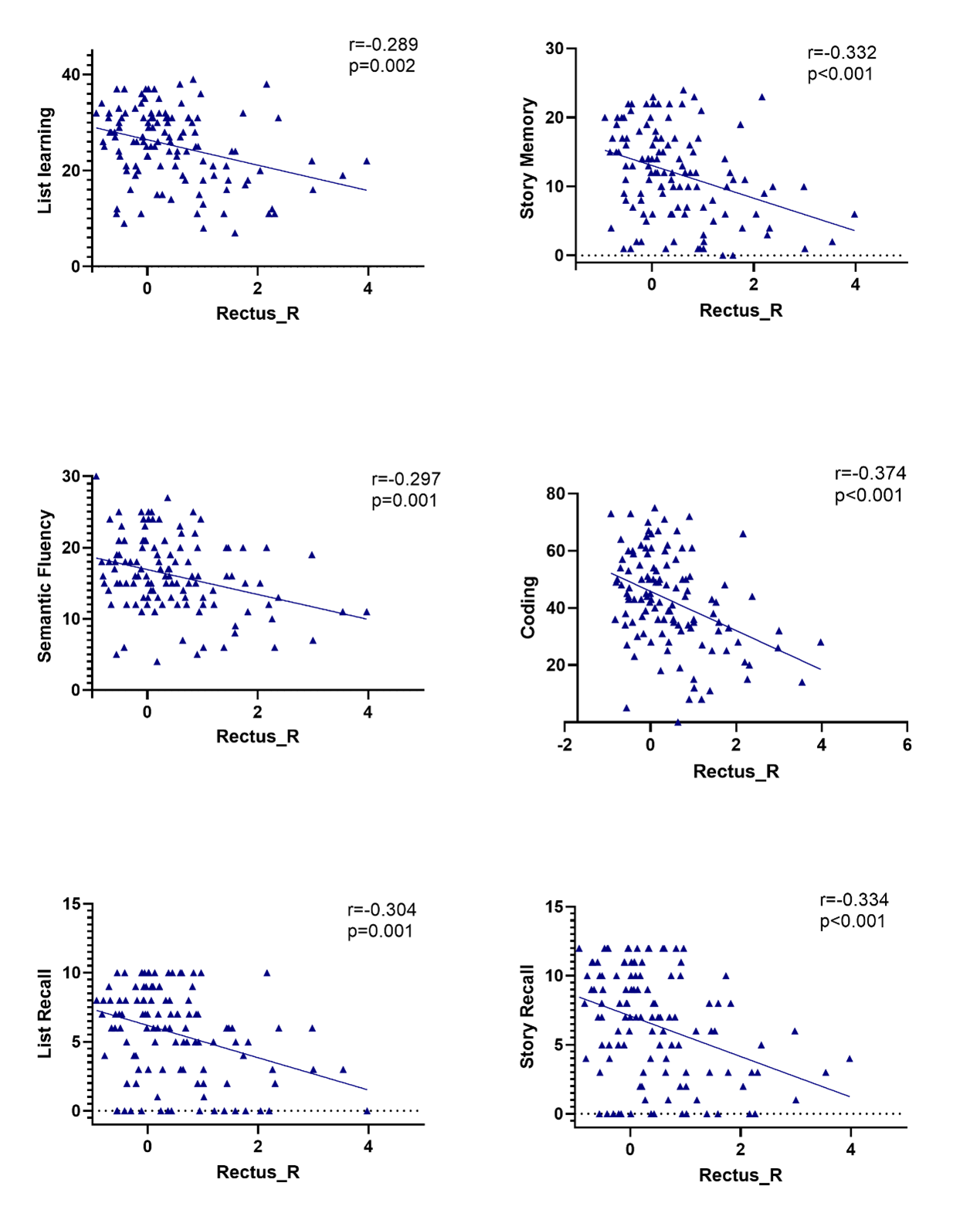


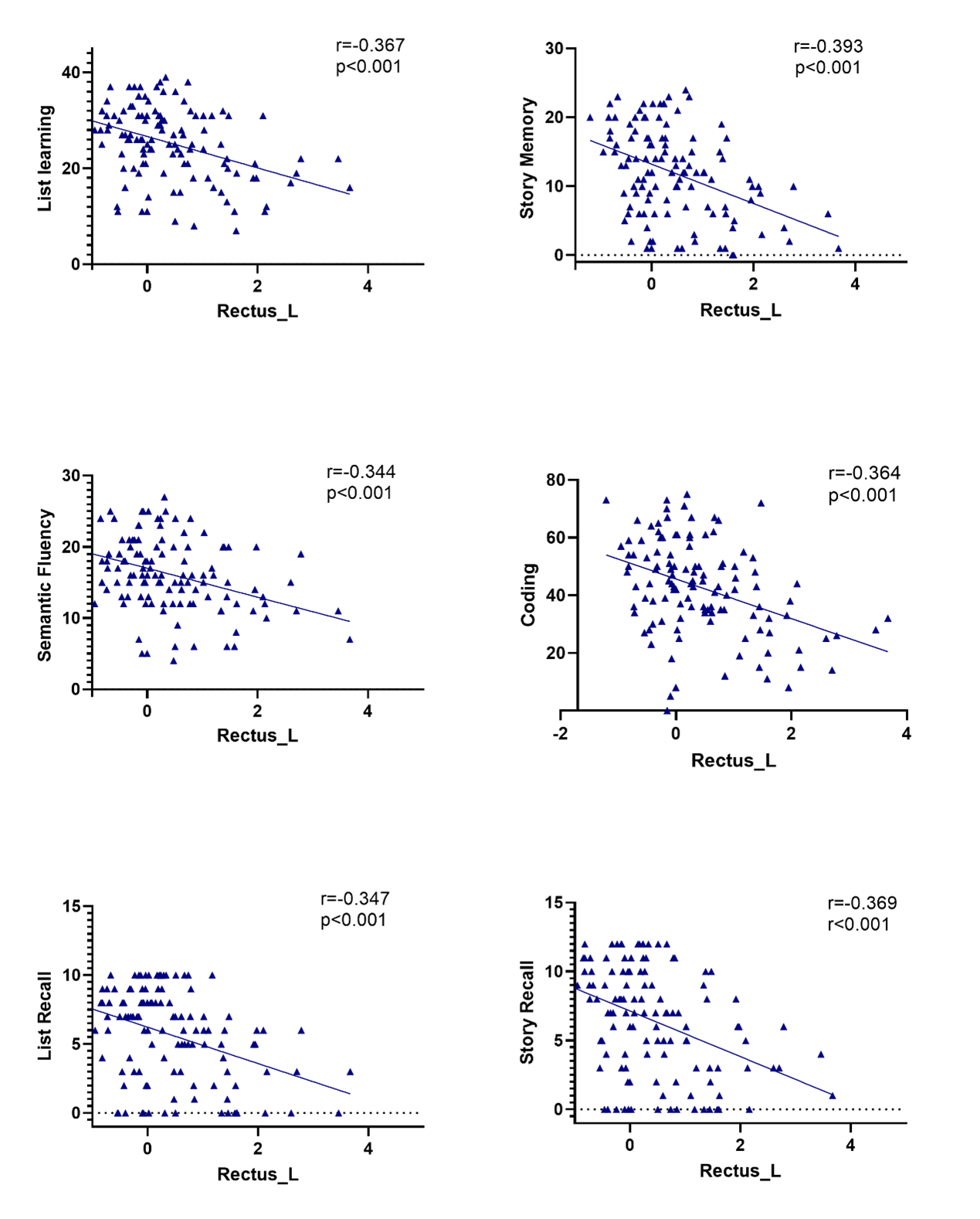


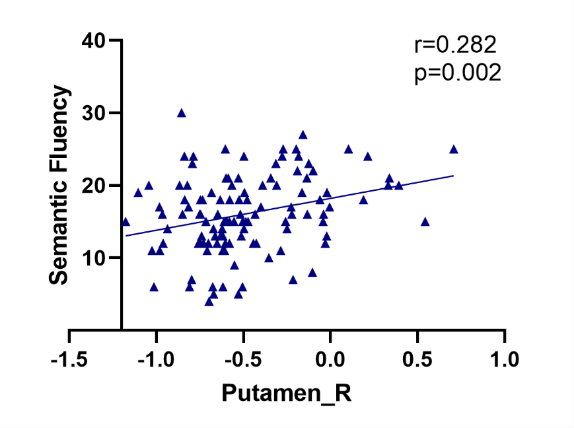


Figure S2: Correlation analysis between slow-5 Rectus_R, Rectus_L, Putamen_R and RBANS scores.
